# In silico genome analysis reveals the metabolic versatility and biotechnology potential of a halotorelant phthalic acid esters degrading *Gordonia alkanivorans* strain YC-RL2

**DOI:** 10.1186/s13568-019-0733-5

**Published:** 2019-02-04

**Authors:** Ruth Nahurira, Junhuan Wang, Yanchun Yan, Yang Jia, Shuanghu Fan, Ibatsam Khokhar, Adel Eltoukhy

**Affiliations:** Biological Laboratory, Department of Biology, Graduate School of Chinese Academy of Agricultural Sciences, Beijing, People’s Republic of China

**Keywords:** *Gordonia*, Aromatic pollutants, Secondary metabolites, Heavy metals, Phthalic acid esters

## Abstract

Members of genus *Gordonia* are known to degrade various xenobitics and produce secondary metabolites. The genome of a halotorelant phthalic acid ester (PAEs) degrading actinobacterium *Gordonia alkanivorans* strain YC-RL2 was sequenced using Biosciences RS II platform and Single Molecular Real-Time (SMRT) technology. The reads were assembled de novo by hierarchical genome assembly process (HGAP) algorithm version 2. Genes were annotated by NCBI Prokaryotic Genome Annotation Pipeline. The generated genome sequence was 4,979,656 bp with an average G+C content of 67.45%. Calculation of ANI confirmed previous classification that strain YC-RL2 is *G. alkanivorans*. The sequences were searched against KEGG and COG databases; 3132 CDSs were assigned to COG families and 1808 CDSs were predicted to be involved in 111 pathways. 95 of the KEGG annotated genes were predicted to be involved in the degradation of xenobiotics. A phthalate degradation operon could not be identified in the genome indicating that strain YC-RL2 possesses a novel way of phthalate degradation. A total of 203 and 22 CDSs were annotated as esterase/hydrolase and dioxygenase genes respectively. A total of 53 biosynthetic gene clusters (BGCs) were predicted by antiSMASH (antibiotics & Secondary Metabolite Analysis Shell) bacterial version 4.0. The genome also contained putative genes for heavy metal metabolism. The strain could tolerate 1 mM of Cd^2+^, Co^2+^, Cu^2+^, Ni^2+^, Zn^2+^, Mn^2+^ and Pb^2+^ ions. These results show that strain YC-RL2 has a great potential to degrade various xenobiotics in different environments and will provide a rich genetic resource for further biotechnological and remediation studies.

**Electronic supplementary material:**

The online version of this article (10.1186/s13568-019-0733-5) contains supplementary material, which is available to authorized users.

## Introduction

Environmental pollution has become a global concern that requires concerted effort for monitoring, control and remediation plans across all sectors of society. Industrialization and urban development, advanced and commercial agriculture have all accelerated pollution. Recalcitrant organic xenobiotic compounds such as polychlorinated biphenyls (PCBs), plasticizers, synthetic polymers have attracted attention not only due to their persistence in the environment but all also their toxicity (El-Shahawi et al. 2010; Schäfer et al. 2011). Metal pollution is another concern for terrestrial and aquatic ecosystems (Fu and Wang 2011). Metals are naturally occurring in earth’s crust but anthropogenic activities have resulted in elevated levels that are detrimental to life. Metals at the right concentrations play crucial roles in biochemical and physiological activities but elevated levels can lead to an equilibrium shift leading to abnormalities and disease (Tchounwou et al. 2012; Wang and Shi 2001). Therefore, there is an urgent need for bioremediation and microbes have a rich potential for remediation of contaminated sites and synthesis of secondary metabolites that would be eco-friendly alternatives to their synthetic congeners (Das and Chandran 2011; Megharaj et al. 2011).

Members of phylum *Actinobacteria* possess a great potential for remediation of contaminated environments through degradation of xenobiotics, sequestration of heavy metals and production of novel secondary metabolites. In the phylum *Actinobacteria*, the genus of *Gordonia* has recently gained attention due to the biotechnological potential of its members (Drzyzga 2012). Members of genus *Gordonia* are high G+C gram positive bacteria of the order *Actinomycetales* (Arenskötter et al. 2004). Their ability includes but is not limited to degradation of various phthalic acid esters (PAEs), poly aromatic hydrocarbons (PAHs), alkylpyridines, 1,3,5-triazines and synthetic isoprene rubber (Drzyzga 2012). Production of extracellular polysaccharides (Ta-Chen et al. 2008), carotenoids (Arenskötter et al. 2004), steroids (Schneider et al. 2008), and surfactants (Drzyzga 2012) has been documented. With major technological advances in genome sequencing, prediction and elucidation of the biosynthetic pathways of these novel compounds through genome mining is now possible. Varied microbial natural products such as polyketide synthases (PKSs, NRPSs, and the ribosomally synthesized and post-translationally modified peptides (RiPPs) (Letzel et al. 2014; Liu et al. 2016) have recently gained attention in the pharmaceutical industry. Most *Gordonia* species contain genes for polyketide and carotenoid biosynthesis and alkane degradation (Sowani et al. 2018).

A halotorelant PAEs degrading *Gordonia alkanivorans* strain YC-RL2 was previously isolated from contaminated soil. It had an outstanding ability to utilize PAEs and other aromatic compounds as the sole carbon and energy source in varied environmental conditions (Nahurira et al. 2017). Strain YC-RL2 could transform dialkyl phthalates (DAPs) into phthalate (PA) via monoalky phthalates (MAPs) and then to benzoic acid (BA). Typically, the biodegradation process of PAEs is initiated by the hydrolysis of ester linkage forming monoester and subsequently PA and then protocatechuate. Though PA degrading genes are well characterized, not so much is known about genes and enzymes involved in the hydrolysis of PAEs to PA and its transformation to benzoate under aerobic conditions. Furthermore, not much is known about the genetic basis of YC-RL2 outstanding ability to utilize diversified substrates and adapt to a wide range of environmental conditions. This study focused on analyzing genome information to unveil the underlying genetic information of strain YC-RL2’s ability to degrade various aromatic compounds, PAEs, synthesize secondary metabolites and survive in contaminated environments.

## Materials and methods

### Chemicals and media

Chemicals used in this study were all of analytical grade. Diphenyl ether, napthol, benzene, *para*-chlorobenzoic acid (PCBA), triphenyl phosphate (TPhP) and phenyl phosphate were purchased from Shanghai Macklin Biochemical Co., Ltd (Shanghai, China). Dichloromethane (CHCl_2_), *o*-xylene, phenol, hexane and naphthalene were Sinopharm Chemical reagent Co., Ltd (Beijing, China). Tetra chlorobenzene and CHCl_3_ were from Beijing Chemical works Ltd while cholesterol was obtained from Beijing Solarbio life technology Co., Ltd (Beijing, China). Biphenyl, PCB No. 1 (2-Chlorobiphenyl), PCB No. 11 (3, 3′-Dichlorobiphenyl), PCB No.31 (2,4′,5-Trichlorobiphenyl), PCB No. 47 (2,2′,4,4′-Tetrachlorobiphenyl) were obtained as standards from Dr. Ehrnstorfer GmbH Laboratories (Augsburg-Germany).

### Substrate utilization tests and intermediate analysis

The ability of strain YC-RL2 to utilize diphenyl ether, naphthol, CHCl_2_, benzene, phenol, PCBA, TPhP, *o*-xylene, hexane, tetra chlorobenzene, cholesterol, naphthalene, CHCl_3_ and phenyl phosphate, biphenyl and PCBs was tested by inoculating the strain in trace element medium (TEM) medium supplemented with 50 ppm of each. Each experiment was setup in triplicate and the setup without inoculum acted as a control treatment. Cell growth was measured with UV–Vis spectrometer (Biomate 3S-Thermoscientific) as optical density at 600 nm after 7 days.

### DNA extraction, genome library preparation and sequencing

The strain was previously isolated from petroleum contaminated soil as described before (Nahurira et al. 2017). It was cultured in LB liquid medium for 4 days at 30 °C and 180 rpm until the OD_600_ reached 1.0. This culture was then used for DNA extraction.

The genomic DNA was extracted using Qiagen QIAamp DNA Kit according to manufacturer’s instructions. The size, quantity and quality of the DNA were checked by Agilent 2100 Bioanalyzer (Santa Clara, CA, USA). The genome was then sequenced by Pacific Biosciences RS II platform and Single Molecular Real-Time (SMRT) technology (Ardui et al. 2018; Shin et al. 2013). The raw sequence data were filtered, and 186,554 high quality reads were obtained with a total of 1,912,577,568 bp and average reads length of 10,252 bp. The reads were assembled de novo by applying the hierarchical genome assembly process (HGAP) algorithm version 2 (Jayakumar and Sakakibara 2017). Prediction and annotation of genes, protein coding sequences, tRNA and rRNA carried out using by NCBI Prokaryotic Genome Annotation Pipeline (https://www.ncbi.nlm.nih.gov/genome/annotation_prok/).

### Phylogenetic and comparative genomic analysis

Strain YC-RL2 was originally classified as *G. alkanivorans* based on the partial sequence of 16S rRNA gene sequence (KR819397.1). To confirm its classification status, the genome was used to calculate Average Nucleotide Identity (ANI) values. ANI values among *Gordonia* strains were calculated by JSpecies (http://jspecies.ribohost.com/jspeciesws/) equipped with local access to BLAST+ and MUMmer. Genome-derived 16S rRNA gene sequence was also used to ascertain its phylogenetic relationship with *Gordonia* strains validly published at LSPN bacterio.net. The sequences were imported to MEGA 6.0 software (Tamura et al. 2013) and analyzed by neighbor-joining algorithm with 1000 bootstrap values.

Genome annotated dioxygenases were searched against SMART (http://smart.embl-heidelberg.de/), interproscan (https://www.ebi.ac.uk/interpro/) and conserved domain database (CDD) (https://www.ncbi.nlm.nih.gov/cdd) to check for the resemblance of domains and filter out false positives. The filtered putative dioxygenases sequences were then aligned using blastP with sequences from swissprot and pdb databases. The alignments producing Expect (E) values of < 10^−10^ were selected for further analysis. In case of alignments with values of > 10^−10^, percentage identity of 30% was used as a criterion. If neither of the above were satisfied, sequences of bitscores of > 50 were considered. The protein sequences were retrieved from NCBI in FASTA format and phylogenetic trees were constructed by neighbor-joining algorithms in the MEGA 6.0 program.

All CDS annotated as esterases/carboxylesterases and hydrolases were compared to the phthalate hydrolases in literature by examining their conserved domains (https://www.ncbi.nlm.nih.gov/cdd) and motifs. The sequences were searched for the signature pentapeptide (GXSXG) and HGG motif that are characteristic of esterases (Hausmann and Jaeger 2010).

### Prediction of secondary metabolite potential

To predict anabolic potential of strain YC-RL2, putative biosynthetic gene clusters (BGCs) were annotated by antiSMASH (antibiotics & Secondary Metabolite Analysis Shell 4.0) (Weber et al. 2018).

### Heavy metal tolerance tests

Heavy metal tolerance was determined by growing the strain aerobically in stationary 96 well plates in LB medium supplemented with appropriate concentration of heavy metals. The growth of the strain was measured by monitoring the changes in the optical density (OD_600_) using an infinite M200PRO (TECAN) plate reader every 24 h for 4 days. All experiments were conducted in triplicate. The strain was tested for growth in divalent heavy metals such as 1 mM of Cd^2+^, Co^2+^, Cu^2+^, Ni^2+^, Zn^2+^, Mn^2+^ and Pb^2+^ ions and 0.1 mM of Hg^2+^. A growth of OD > 0.2 was considered as metal resistant for that treatment (Uhrynowski et al. 2017).

### Accession numbers

Strain YC-RL2 has been deposited in China General Microbiological Culture Collection Center (CGMCC) under accession number CGMCC 10992. The complete chromosome and plasmid sequences of *G. alkanivorans* strain YC-RL2 were deposited in GenBank database under accession number CP027114 and CP027115 respectively. The BioProject and BioSample information is available at PRJNA434635 and SAMN08565628 respectively.

## Results

### Genome sequence and annotation results

The generated genome sequence of strain YC-RL2 comprised of 4,979,656 bp with an average G+C content of 67.45%. The sequence was assembled into one circular chromosome (4,921,996 bp and one plasmid (pYC01, 57,660 bp). In total, 4541 genes were predicted, including 4308 protein coding sequences with 63 rRNA genes. More genome features are presented in Table 1.

**Table 1:** A table showing genome features of YC-RL2

Gene features	Number/comment
Genes (total)	4541
CDS	4478
CDS (coding)	4308
Genes (RNA)	63
RNA genes	63
rRNAs	4, 4, 4 (5S, 16S, 23S)
tRNAs	48
ncRNAs	3
Pseudo genes (total)	170
Pseudo genes (ambiguous residues)	0 of 170
Pseudo genes (frame shifted)	79 of 170
Pseudo genes (incomplete)	97 of 170
Pseudo genes (internal stop)	20 of 170
Pseudo genes (multiple problems)	24 of 170

### Classification

The strain was originally classified as *G. alkanivorans* strain YC-RL2 based on its partial 16S rRNA gene sequence (KR819397.1). With the genome results, we aligned the 16S rRNA gene sequence against the 39 *Gordonia* strains validly published at LSPN bacterio.net. The strain YC-RL2 was closely related with *G. nitida* (AF148947.1) though clustered together with *G. alkanivorans* (Y18054.1) (Additional file 1: Figure S1). Using BLAST, the complete 16S rRNA gene sequence of strain YC-RL2 showed 100% identity with 16S rRNA gene sequence of AB065369.1, *G. alkanivorans* strain CC-JG39 (AY864338.1), *Gordonia* sp. CNJ863 PL04 (DQ448700.1) and *G. alkanivorans* strain DSM 44187 (AY995556.1).

We therefore calculated the average nucleotide identity (ANI) with JSpecies (http://jspecies.ribohost.com/jspeciesws/). ANI is a similarity index between a given pair of genomes that can be applicable to prokaryotic organisms independently of their G+C content. The calculated ANIb, ANIm and Pearson correlation coefficient values of strain YC-RL2 and *G. alkanivorans* NBRC 16433 and *G. alkanivorans* CGMCC 6845 were: 97.03% and 96.71%, 97.91% and 97.56%, 0.99974 and 0.99964 respectively. Though *G. rubripertincta* NBRC 101908 shows a Pearson correlation coefficient greater than the cutoff of 0.999, its ANib and ANIm results were below the cut-off of 95% (Table 2). These findings support the initial classification that strain YC-RL2 belongs to *G. alkanivorans* species according to cut-off values for species differentiation (95–96% for ANI).

**Table 2:** A table showing ANI values as calculated by ANIb and ANIm

Genome	ANIb [%]	ANIm [%]	Pearson correlation coefficient
*Gordonia alkanivorans* NBRC 16433	*97.03*	*97.91*	*0.99974*
*Gordonia alkanivorans* CGMCC 6845	*96.71*	*97.56*	*0.99964*
*Gordonia rubripertincta* NBRC 101908	91.85	92.66	*0.99933*
*Gordonia namibiensis* NBRC 108229	91.29	92.39	0.99896
*Gordonia paraffinivorans* NBRC 108238	82.21	86.1	0.99402
*Gordonia* sp. KTR9	80.62	85.79	0.9925
*Gordonia terrae* NBRC 100016	80.48	85.71	0.98842
*Gordonia bronchialis* DSM 43247	76.56	84.95	0.97643
*Gordonia soli* NBRC 108243	75.21	84.68	0.96966
*Gordonia amarae* NBRC 15530	74.95	84.67	0.95839
*Gordonia sputi* NBRC 100414	74.62	84.6	0.95702
*Gordonia aichiensis* NBRC 108223	74.35	84.6	0.94806
*Gordonia* sp. QH-11	73.31	84.55	0.92141
*Gordonia sihwensis* NBRC 108236	73.08	84.44	0.90709

Italicized values show results above the cut-off points

### COG analysis and KEGG analysis

The CDSs were searched against the KEGG and COG databases to investigate the functions of the genes and metabolic pathways they are involved in (Kanehisa et al. 2018). 3132 CDSs were allocated to COG families and 1808 CDSs were involved in 111 pathways.

**Fig. 1:**
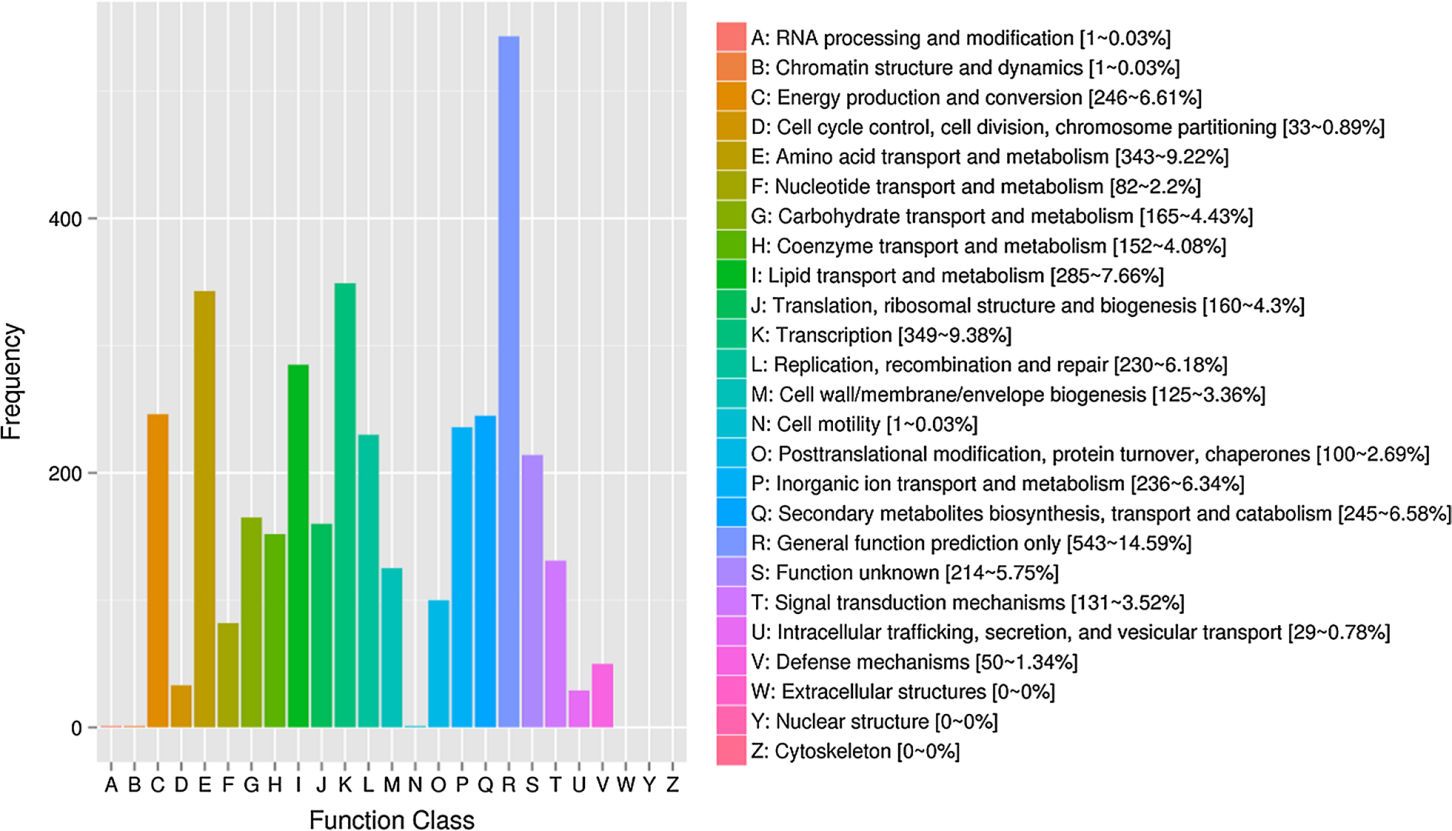
COG analysis of YC-RL2 genome. The X-axis represents the COG function class; the Y-axis is the number of genes. Percentage of genes in different classes reflects the metabolic or physiological bias in the corresponding period and environment

The database of Clusters of Orthologous Groups of proteins (COGs) phylogenetically classifies proteins (http://www.ncbi.nlm.nih.gov/COG) by applying an exhaustive comparison of all protein sequences from genomes (Tatusov 2000). The result of COG analysis of YC-RL2 as shown in Fig. 1 and the circular representation of the genome was further visualized by Circos (Krzywinski et al. 2009) as displayed in Additional file 1: Figure S2. The COG database provides a platform to assess evolutionary-oriented relationships of protein families. A large portion of CDS (1394) were not annotated to any COG groups.

95 of the KEGG annotated genes were predicted to be involved in the degradation of xenobiotics such as chloroalkanes, chloroalkenes, chlorobenzene, benzoate, flourobenzoate, dioxins, *o*-xylene, toluene, naphthalene, styrene and atrazine. 26 more genes were annotated to be involved in aromatic compound degradation.

**Fig. 2:**
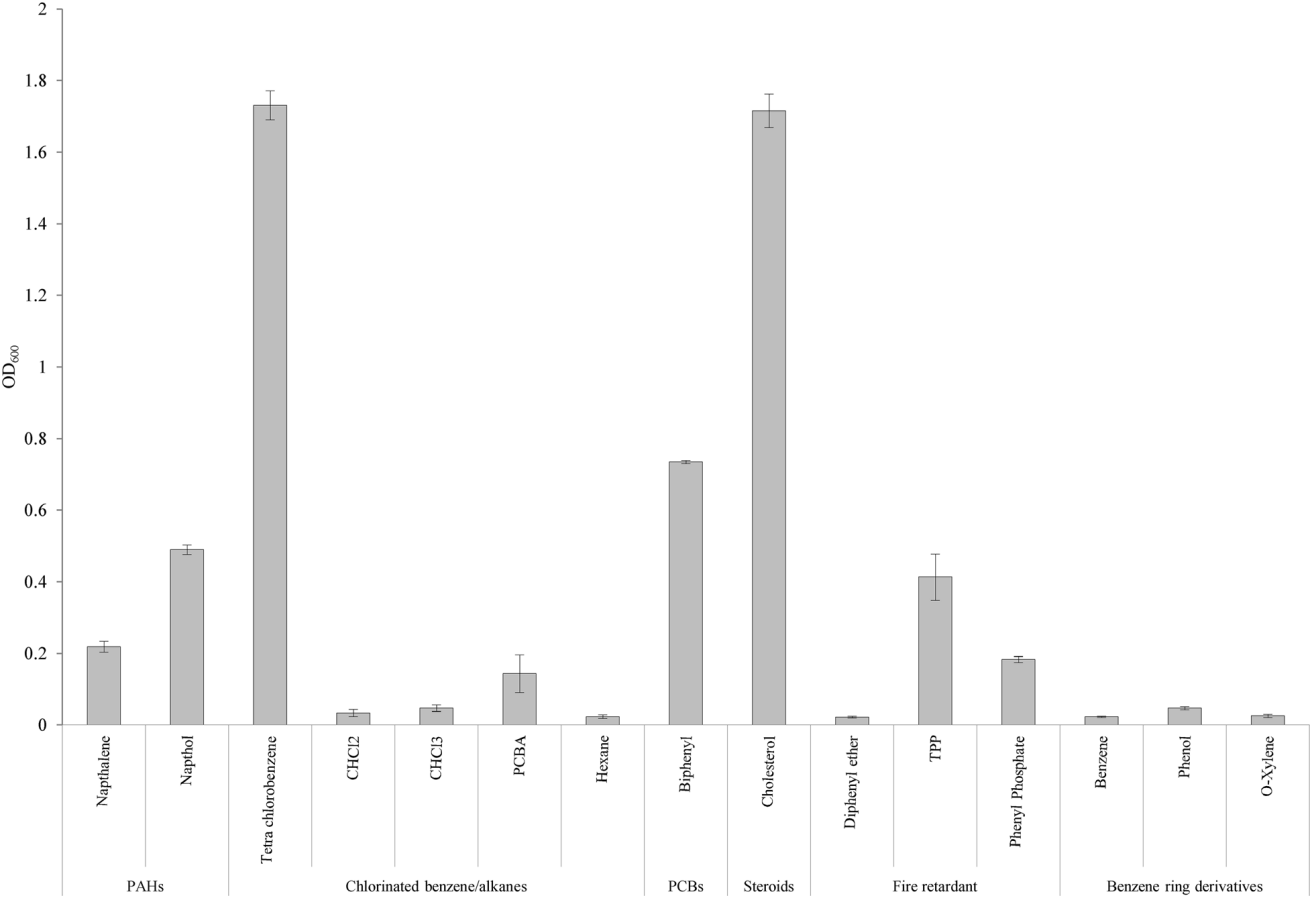
A figure showing the growth of YC-RL2 when inoculated with different substrates as sole carbon and energy source. The OD_600_ measurements were taken after 7 days of incubation. The experimental set up consisted of TEM medium with 1% (V/V) of YC-RL2 inoculum and appropriate amount of substrates. The abiotic setup was used as a control. For each setup, it was conducted in triplicate and errors show standard deviation from the mean value

**Fig. 3:**
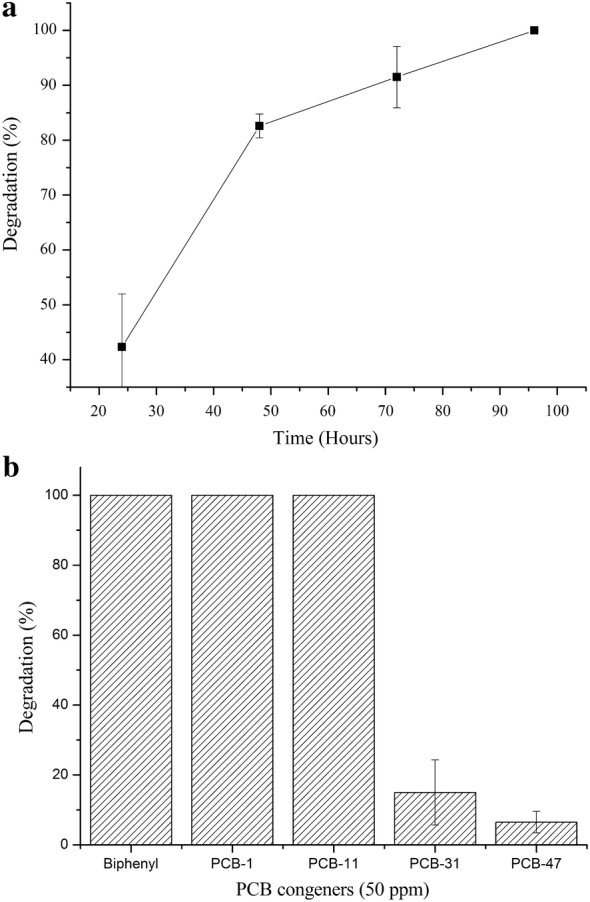
A figure showing **a** the ability of YC-RL2 to degrade biphenyl (BP), **b** ability of YC-RL2 to degrade selected PCB congeners (50 ppm). The reaction was setup using TEM medium using BP, PCB congeners as the sole carbon and energy source in each case. The inoculum was 1% (V/V) of the medium. After 7 days of incubation (for PCB congener experiments), the residues were quantified using HPLC. Each setup was done in triplicate and error bars represent standard deviation from the mean value

From the KEGG prediction, we tested the ability of strain YC-RL2 to utilize diphenyl ether, naphthol, dichloromethane, benzene, phenol, PCBA, tri phosphate (TPhP), *o*-xylene, hexane, tetra chlorobenzene, cholesterol, naphthalene, CHCl_3_ and phenyl phosphate as the sole carbon and energy source and the results are shown in the Fig. 2. The cell growth was measured with UV–Vis spectrometer as optical density at 600 nm. We also tested the strain’s ability to degrade various PCBs and the results are shown in Fig. 3. To the best of our knowledge, this is the first report of a *Gordonia* species to degrade PCBs. The position and number of chlorine substitutions affect biodegradability of PCBs. Strain YC-RL2 could easily degrade biphenyl, PCB-1 and PCB-11 but couldn’t degrade PCB-31, PCB-47 and other highly substituted PCBs.

### Analysis of phthalate catabolic genes

#### Putative PAEs esterases

A total of 203 CDS were annotated as esterases or hydrolases in the genome of strain YC-RL2. Esterases hydrolyze a broad spectrum of substrates (Ren et al. 2016), demonstrating that strain has great potential for degrading various xenobiotics.

**Fig. 4:**
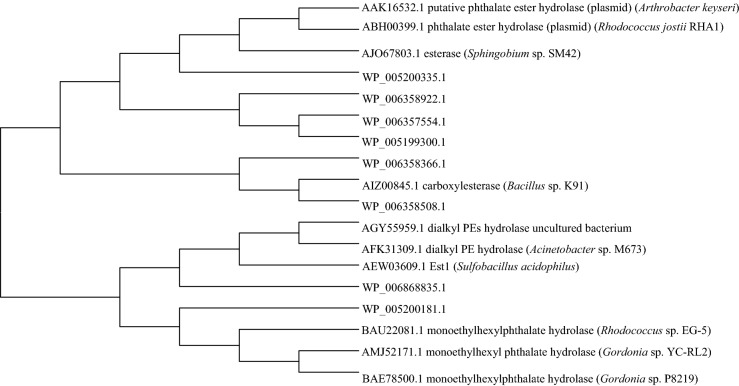
A phylogenetic tree showing the relationship between selected putative esterases/hydrolases in YC-RL2 genome and reported phthalate hydrolases. The evolutionary history was inferred using the neighbor-joining algorithm. The bootstrap consensus tree inferred from 1000 replicates is taken to represent the evolutionary history of the taxa analyzed. The codes used for the sequences are Refseq accession numbers as annotated by NCBI

We compared genome derived esterases/hydrolase sequences to those of reported PAEs hydrolases in literature. We chose those that shared conserved domains with the reported hydrolases. Phthalate hydrolases in literature have *para*-nitrobenzyl (PnbA), pimeloyl-methyl ester esterase (MhpC), acetyl ester (Aes), *N*-Carbamoylsarcosine amidohydrolase (CSHase) and *β*-lactamase domains. The genome of strain YC-RL2 possesses 2 PnbA, 20 MhpC, 3 Aes and 3 *β*-lactamase domain-containing putative esterases. The MhpC domain was found in monoethylhexyl phthalate hydrolases (Iwata et al. 2015; Nahurira et al. 2018) and the other domains in DAP hydrolases. A monoethylhexyl phthalate hydrolase (MehpH) was cloned from strain YC-RL2 and characterized (Nahurira et al. 2018). This enzyme was able to convert MAPs to PA. Eight putative esterases were considered for further analysis (denoted by Refseq accessions of protein product as annotated by NCBI). Though they shared conserved domains with the reported DAP hydrolases, they showed low identity: WP_006357554.1 shared 26% with CarEW from *Bacillus* sp. K91(Ding et al. 2015), WP_005199300.1 and WP_006868835.1 showed 26% and 32% respectively with Est1 from *Sulfobacillus acidophilus* (Zhang et al. 2014), WP_005200335.1, WP_006358922.1 and WP_005200181.1 shared 26%, 27% and 38% respectively with EstG from *Sphingobium* sp. SM42 (Whangsuk et al. 2015), WP_006358366.1 and WP_006358508.1 shared 35% and 37% with CarEW from *Bacillus* sp. K91 respectively. A phylogenetic tree showing the relationship of the putative esterases/hydrolases and reported DAP hydrolases is shown in Fig. 4.

#### Putative decarboxylases

Analysis of PAEs degradation intermediates showed that mono ethylhexyl phthalate (MEHP), PA and benzoic acid (BA) were the major intermediates (Nahurira et al. 2017). We therefore hypothesized that PA could be decarboxylated to BA. Ebenau-Jehle et al. (2017) reported the role of phthalate and succinyl-CoA-specific CoA transferase and a UbiD-like phthaloyl-CoA decarboxylase in anaerobic phthalate degradation (Ebenau-Jehle et al. 2017). Zhao et al. (2018) also predicted that an aerobic bacterium, *Rhodococcus* sp. 2G, could decarboxylate PA to BA through phthaloyl-CoA and finally to benzoyl-CoA (Zhao et al. 2018). We therefore searched phthaloyl-CoA decarboxylase and succinyl-CoA and phthalate-specific CoA transferase sequences against the genome of YC-RL2. However, there were no decarboxylases that shared similarity to those reported. This is not surprising because strain YC-RL2 is an obligate aerobe and is most likely to lack anaerobic enzymes.

However, a total of 16 decarboxylases were predicted in the genome of YC-RL2 and these were annotated as aspartate, carboxymuconolactone, acetoacetate, threonine-phosphate, uroporphyrinogen, orotidine-5′-phosphate, bifunctional phosphopantothenoylcysteine, diaminopimelate, multifunctional oxoglutarate and OHCU decarboxylases.

#### Benzoate catabolism operon

**Fig. 5:**
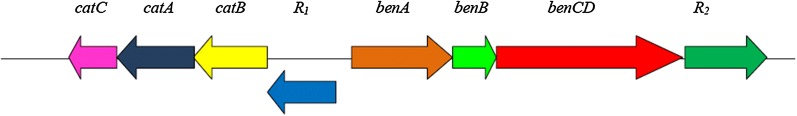
A figure showing the arrangement of benzoate (*benABCDR*_*2*_) and catechol (*catCABR*_*1*_) operons in strain YC-RL2 (Not drawn to scale) as annotated by RAST

Analysis of YC-RL2 genome sequence revealed several gene clusters involved in degradation of organic compounds. Complete operons involved in the degradation of biphenyl, benzoate and catechol were annotated in the genome. Analysis of the putative benzoate operon by Rapid Annotation using Subsystems Technology (RAST) version 2.0 (Aziz et al. 2008; Brettin et al. 2015) revealed that the deduced amino acid sequences encoded by the YC-RL2 benzoate catabolic genes are arranged in the order *benABCD* (Fig. 5). The genes *benC* and *benD* are fused together with a C-terminal ligand binding (FCD) domain (Additional file 1: Figure S3) characteristic of GntR regulatory proteins.

#### Diversity of dioxygenases

**Fig. 6:**
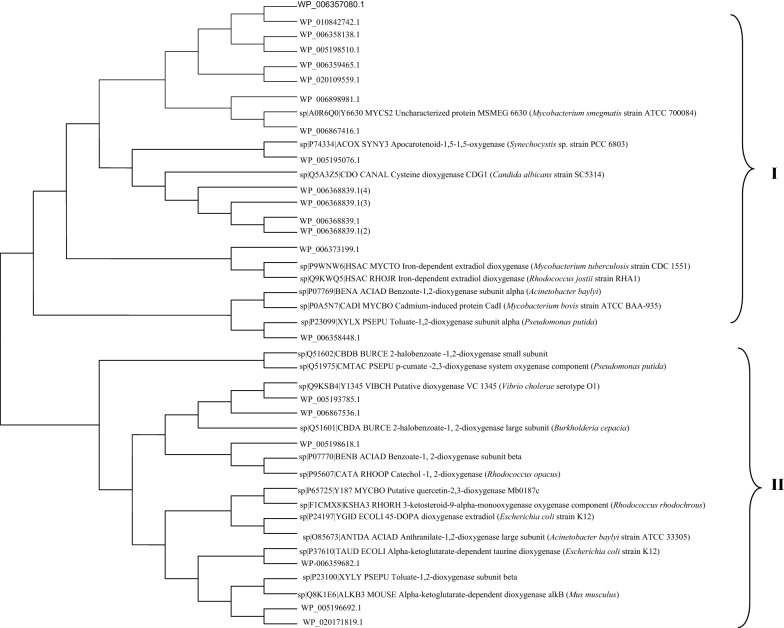
A phylogenetic tree showing the relationship between genome annotated dioxygenases and related dioxygenases. Dioxygenases from YC-RL2 were denoted using the RefSeq accession number of the protein product as annotated by NCBI. The tree was drawn in MEGA 6.0 by neighbor joining algorithm and further annotated by iTOL version 3

A total of 22 dioxygenases were annotated in the genome. These sequences were checked with interpro, CDD and SMART databases to filter out false positives. Conserved domains as predicted by SMART and CDD are shown in Additional file 1: Table S1. Two sequences annotated as nitropropane dioxygenases were not considered for further analysis as that classification has been rendered obsolete and are currently classified as nitronate monooxygenases. The sequences were aligned using BlastP in the swissprot and pdb databases and retrieved sequences were used to construct a phylogenetic tree by MEGA 6.0 software and further annotated by iTOL (Letunic and Bork 2016). The phylogenetic tree showed that dioxygenases are clustered as shown in Fig. 6. Most dioxygenases were classified as ring cleaving or hydroxylating based on the swissprot or pdb and conserved domain database.

#### Secondary metabolite biosynthesis potential

To predict anabolic potential of strain YC-RL2, putative biosynthetic gene clusters (BGCs) were annotated by antiSMASH (antibiotics & Secondary Metabolite Analysis Shell 4.0) (Weber et al. 2018). A total of 53 gene clusters were predicted; 6 non-ribosomal peptide synthetases (Nrps), 2 terpene, 1 arylpolyene, 1ectoine, 1 Type 1 polyketide synthases (T1pks), 1 bacteriocin, 1 fatty acid and 39 putative gene clusters (Additional file 1: Table S2). Such similar gene clusters were reported in *G. lacunae* BS2^T^ but have not been experimentally determined (Durrell et al. 2017).

#### Heavy metal resistance

The genome of strain YC-RL2 contains putative genes for heavy metal transport and resistance such as mercury (II) reductase, copper resistance proteins CopD and CopC, arsenical-resistance protein and tellurium resistance protein TerC. Amino acid sequences of genome derived metal resistance genes were searched against BacMet (Antibacterial Biocide and Metal Resistance Genes Database) using default parameters (Pal et al. 2014). The putative genes are located on genomic islands as predicted by IslandViewer 4 (Bertelli et al. 2017) (data not shown).

**Fig. 7:**
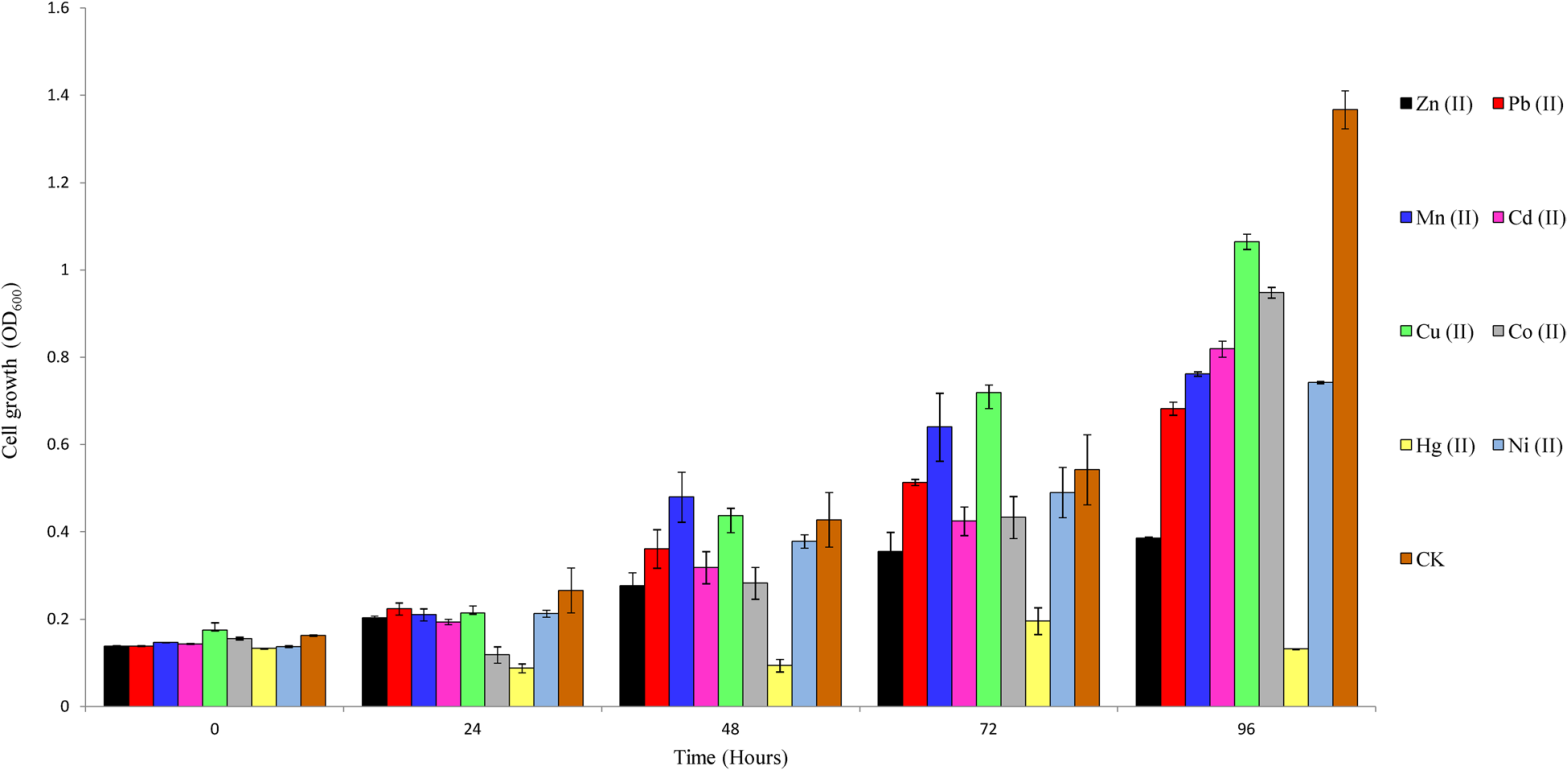
A graph showing tolerance of strain YC-RL2 to heavy metal ions. The strain was inoculated in LB liquid medium supplemented with and tolerance of 1 mM of Cd^2+^, Co^2+^, Cu^2+^, Ni^2+^, Zn^2+^, Mn^2+^ and Pb^2+^ ions and 0.1 mM of Hg^2+^. Two controls were set up for each treatment. The setup with metal ions but no inoculum was considered as negative control and was used as a blank measurement. The inoculum without metal ions was considered as the positive control and as is denoted as CK. The experiment was done in triplicate for each heavy metal ion tested. The mean values were used to plot the graph and error bars represent standard deviation from the mean

The strain was tested for growth in and tolerance of 1 mM of Cd^2+^, Co^2+^, Cu^2+^, Ni^2+^, Zn^2+^, Mn^2+^ and Pb^2+^ ions and 0.1 mM of Hg^2+^ and the result is shown in Fig. 7. The strain was tolerant to heavy metals tested except Hg^2+^.

## Discussion

From the results of 16S rRNA alignment and ANI calculation, strain YC-RL2 could be classified as *G. alkanivorans*. With a plethora of prokaryotic genomes in public databases, genomic classification was proposed by Chun et al. (2018) as the most reproducible and reliable method to infer phylogenetic relationships among prokaryotes (Chun et al. 2018).

The aerobic degradation pathway of PAEs in bacteria is initiated by the hydrolysis of ester bond to form corresponding MAPs. MAPs are further hydrolyzed to form PA (Liang et al. 2008) and esterases are the key enzymes (Luo et al. 2012). PA is then completely mineralized via protocatechuate (Benjamin et al. 2015) which is a central intermediate of aromatic ring degradation.

A previous study involving strain YC-RL2 showed that the strain could degrade di-2-ethylhexyl phthalate (DEHP) to PA and then BA instead of protocatechuate (Nahurira et al. 2017). More so, a phthalate degradation operon could not be identified in the genome of YC-RL2. We therefore hypothesized that PA could be decarboxylated to BA. Similar pathways were reported in *Microbacterium* sp. CQ0110Y (Chen et al. 2007), *Mycobacterium* sp. YC-RL4 (Ren et al. 2016). Thus the genome of strain YC-RL2 would have benzoate and catechol degradation clusters. We therefore analyzed the diversity of esterases, dioxygenases and decarboxylases. The putative esterases (Fig. 4) clustered majorly into two groups though the group containing CarEW from *Bacillus* sp K91was distinct. This is the only reported PAE esterase capable of hydrolyzing two bonds. This group contained WP_006358366.1 and WP_006358508.1 both annotated as carboxylesterases.

In typical phthalate degradation, further steps are accomplished by dioxygenases, dehydrogenases and later decarboxylases. A total of 22 dioxygenases were annotated in the genome and the phylogenetic tree between related dioxygenases shows that they are clustered into two major groups; Group I and II. The benzene ring is the most widely spread and thermodynamically stable chemical moiety which leads to its persistence in nature. Therefore, many aromatic compounds are major environmental pollutants (Díaz 2004). Ring cleavage is a crucial step in the aerobic degradation of aromatic pollutants and this is effected by oxygenases especially dioxygenases. Aromatic ring hydroxylating dioxygenases incorporate two atoms of molecular oxygen into their substrates to form *cis*-1, 2-dihydroxycyclohexadiene intermediates that are further acted upon by *cis*-diol dehydrogenases. This group of dioxygenases includes toluene, benzene, phthalate, naphthalene and biphenyl dioxygenases which are very important in xenobiotics degradation. Ring-cleaving dioxygenases on the other hand, catalyze fission of catecholic compounds. No dioxygenases showed similarity to reported phthalate dioxygenases and a complete phthalate operon could not be annotated in the genome. This shows that strain YC-RL2 possesses a unique pathway of phthalate degradation that needs to be explored further.

There were complete catechol and benzoate gene clusters annotated in the genome. The gene organization of YC-RL2 *benABCD* operon differs from that of most reported *benABCD* operons (Ezezika et al. 2006; Kitagawa et al. 2001; Zhan et al. 2008). The genes *benC* and *benD* are fused together with a C-terminal ligand binding domain (FCD) (Additional file 1: Figure S3) characteristic of GntR regulatory proteins. This domain is found in regulators of sugar biosynthesis operons that bind DNA through a helix-turn-helix (HTH) motif. There are two regulator coding genes; one upstream (*R*_*1*_) and other downstream (*R*_*2*_). The regulator R_1_ belongs to LsyR regulators while R_2_ belongs to the LuxR unlike most LsyR regulators common to catechol and benzoate catabolism operons (Collier et al. 1998; Ezezika et al. 2006). LuxR regulators have a characteristic alpha-helical protein domains fold with sequence-specific DNA binding domains. R_1_ showed 35% identity to *ben* and *cat* operon transcriptional regulator from *Acinetobacter baumannii* (SCZ15558.1) (Zhan et al. 2008), 37% to uncharacterized HTH-type transcriptional regulator YnfL (P77559.1) (Aiba et al. 1996) and 33% to HTH-type transcriptional regulator BenM (O68014.2) (Collier et al. 1998). R_2_ also showed 59% to HTH type of regulators Rv0890c (P9WMG1.1) and Mb0914c (P59969.1) which are mycobacterial proteins.

In addition to degradation of xenobiotics, *Gordonia* members are known to be a source of natural products. A total of 53 putative BGCs were predicted in the genome. Predicted Nrps clusters shared little similarity to known gene clusters such as chartreusin (15%), cahuitamycins (12%), kanamycin (2%), echosides (11%). Predicted terpene clusters showed 6% and 62% similarity with SF2575 and sioxanthin biosynthetic gene clusters respectively. Sioxanthin is a class of glycosylated carotenoids reported in the genus of *Salinispora*; marine actinomycetes (Richter et al. 2015). Although the genus of *Gordonia* is well known for carotenoid biosynthesis, no clusters were annotated as carotenoid BGCs. However, 1 arylpolyene and 1Type I PKS cluster were predicted. Arylpolyene are class of pigments that are functionally similar to carotenoids and protect the bacterium from reactive oxidation (Schöner et al. 2016). Arylpolyenes are polyketide derivatives each contain a terminal aryl moiety connected to a polyene carboxylic acid. To our knowledge, arylpolyene production has not been experimentally proven but was also predicted in *G. lacunae* BS2^T^ (Durrell et al. 2017).

Annotated ectoine cluster shared 75% similarity to BGC0000853_c1 ectoine cluster. The predicted ectoine cluster comprised of acetyl glutamate-5-kinase, TetR family transcriptional regulator, haloalkane dehalogenase, ectoine synthase, aminotransferase class III and GCN5 related-acetyltransferase genes. Ectoine is one many compatible solutes that protect and stabilizes cell membranes, proteins and DNA against drying and high temperatures, high osmolarity, and even pollutants (Bursy et al. 2008; Graf et al. 2008; Nakayama et al. 2000; Schnoor et al. 2004). In addition to ectoine biosynthesis, more genes coding for betaine, glycine and trehalose synthesis and transport were predicted in the genome. This explains the halotolerant nature of strain YC-RL2.

Some of the BGCs annotated as putative shared similarity with known BGCs for instance bacillomycin (20%), coelichelin (27%), nataxazole (7%), rifamycin (3%) and paromomycin (5%). The ability to produce diversified secondary metabolites would not only enable the strain to survive in different ecological niches but also be harnessed for therapeutical, industrial and pharmaceutical uses.

Metals have a central role in the life processes of microorganisms. Some metals such as copper, iron, and zinc are essential in biochemical, structural and osmotic processes (Sterritt and Lester 1980). However, high metal concentration can be detrimental to microorganisms by displacement of essential metals from their native binding sites or through ligand interactions. Bacteria adapt to metals through a variety of genome-mediated resistance systems (Hassen et al. 1998; Tchounwou et al. 2012). Some *Gordonia* strains such as *Gordonia* sp WQ-01A, *G. amicalis* HS-11 and *G. amarae* MTCC 4818 are known to reduce some metals by formation of nanoparticles (Sowani et al. 2018).

The present study reveals that strain is metabolically versatile and has a high potential for secondary metabolites can be harnessed for various clinical and industrial applications. Presence of diverse dioxygenases, esterase/hydrolase and metal resistant genes provide insight into the ability of this strain to survive in varied ecological niches. Cloning and expression of esterases involved in PAEs hydrolysis is currently underway. Based on these results, strain YC-RL2 genome will provide a rich genetic resource for further biotechnological and remediation studies.

## Additional file

**Additional file 1: Figure S1.** A phylogenetic tree showing the relationship between 16S rDNA of strain YC-RL2 and 16S rDNA of validly published *Gordonia* species. The evolutionary history was inferred using the Neighbor-Joining algorithm. The bootstrap consensus tree inferred from 1000 replicates is taken to represent the evolutionary history of the taxa analyzed. **Figure S2.** Genome visualization by Circos. **Figure S3.** The conserved domains of putative *ben*CD as predicted by NCBI conserved domain database (https://www.ncbi.nlm.nih.gov/cdd). **Table S1.** The Table showing conserved domains putative dioxygenases in YC-RL2 genome as predicted by SMART and CDD databases. **Table S2.** The table showing BGCs prediction based on the genome sequence of YC-RL2.
