# Supplementary material for: In silico genome analysis reveals the metabolic versatility and biotechnology potential of a halotorelant phthalic acid esters degrading Gordonia alkanivorans strain YC-RL2
Source: AMB Express. 2019 Feb 4;9:21. doi: 10.1186/s13568-019-0733-5 (PMC6362181; doi:10.1186/s13568-019-0733-5)
Supplement: Supplementary file 1 — Additional file 1: Figure S1. A phylogenetic tree showing the relationship between 16S rDNA of strain YC-RL2 and 16S rDNA of validly published Gordonia species. The evolutionary history was inferred using the Neighbor-Joining algorithm. The bootstrap consensus tree inferred from 1000 replicates is taken to represent the evolutionary history of the taxa analyzed. Figure S2. Genome visualization by Circos. Figure S3. The conserved domains of putative benCD as predicted by NCBI conserved domain database (https://www.ncbi.nlm.nih.gov/cdd). Table S1. The Table showing conserved domains putative dioxygenases in YC-RL2 genome as predicted by SMART and CDD databases. Table S2. The table showing BGCs prediction based on the genome sequence of YC-RL2. [file 13568_2019_733_MOESM1_ESM.pdf]

AMB Express

Additional file 1

In silico genome analysis reveals the metabolic versatility and biotechnology potential of a halotorelant phthalic acid esters degrading *Gordonia alkanivorans* strain YC-RL2

Ruth Nahurira[1], Junhuan Wang[1], Yanchun Yan[1*], Yang Jia[1], Shuanghu Fan[1], Ibatsam Khokhar[1], Adel Eltoukhy[1]

[1]Biological Laboratory, Department of Biology, Graduate School of Chinese Academy of Agricultural Sciences, Beijing, P.R. China

*** Correspondence:**

Name: Yanchun Yan

Email: yanyanchun@caas.cn

Telephone: 010-82109685

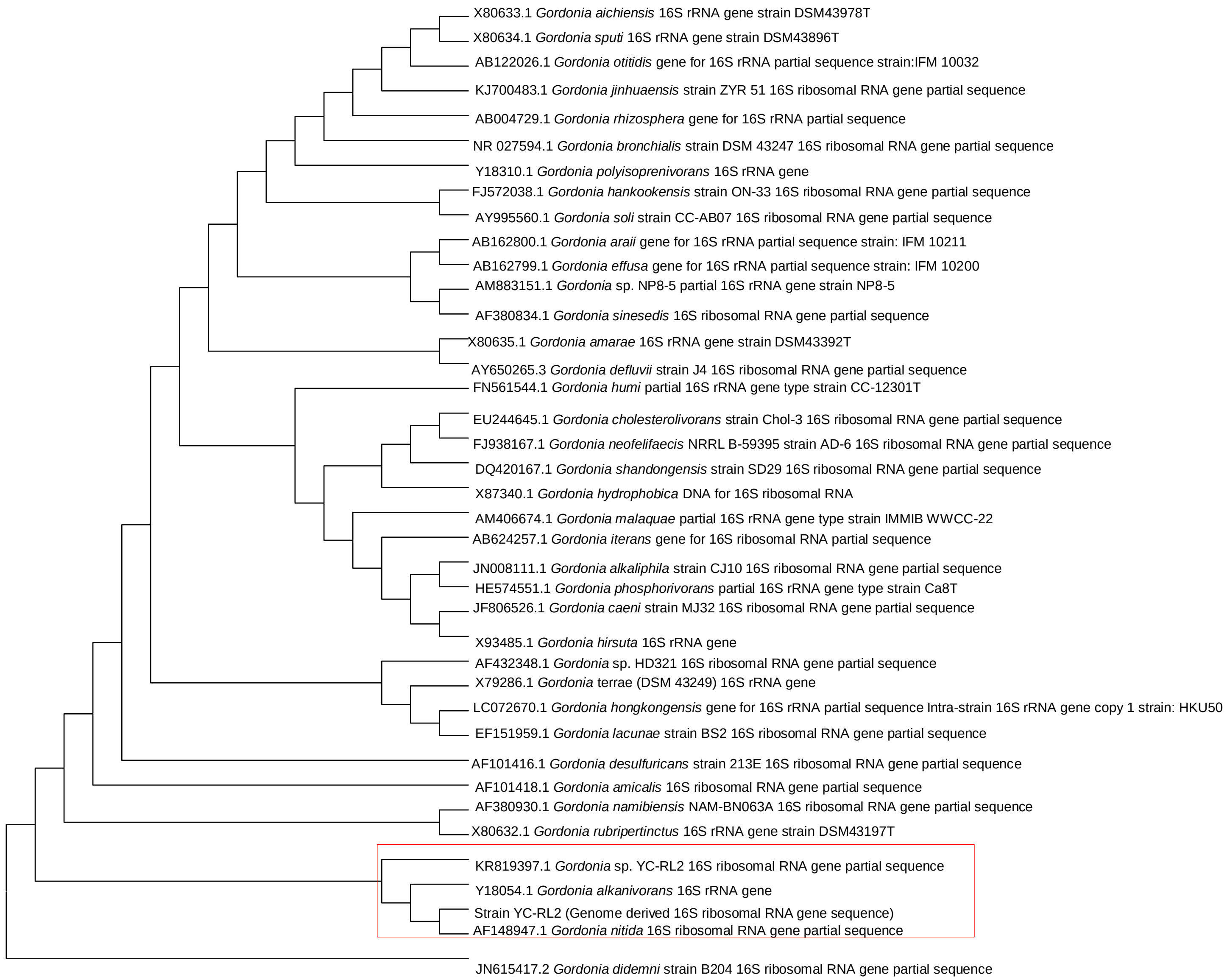
X80633.1 Gordonia aichiensis 16S rRNA gene strain DSM43978T
X80634.1 Gordonia sputi 16S rRNA gene strain DSM43896T
AB122026.1 Gordonia otitidis gene for 16S rRNA partial sequence strain:IFM 10032
KJ700483.1 Gordonia jinhuaensis strain ZYR 51 16S ribosomal RNA gene partial sequence
AB004729.1 Gordonia rhizosphera gene for 16S rRNA partial sequence
NR 027594.1 Gordonia bronchialis strain DSM 43247 16S ribosomal RNA gene partial sequence
Y18310.1 Gordonia polyisoprenivorans 16S rRNA gene
FJ572038.1 Gordonia hankookensis strain ON-33 16S ribosomal RNA gene partial sequence
AY995560.1 Gordonia soli strain CC-AB07 16S ribosomal RNA gene partial sequence
AB162800.1 Gordonia araii gene for 16S rRNA partial sequence strain: IFM 10211
AB162799.1 Gordonia effusa gene for 16S rRNA partial sequence strain: IFM 10200
AM883151.1 Gordonia sp. NP8-5 partial 16S rRNA gene strain NP8-5
AF380834.1 Gordonia sinesedis 16S ribosomal RNA gene partial sequence
X80635.1 Gordonia amarae 16S rRNA gene strain DSM43392T
AY650265.3 Gordonia defluvii strain J4 16S ribosomal RNA gene partial sequence
FN561544.1 Gordonia humi partial 16S rRNA gene type strain CC-12301T
EU244645.1 Gordonia cholesterolivorans strain Chol-3 16S ribosomal RNA gene partial sequence
FJ938167.1 Gordonia neofelifaecis NRRL B-59395 strain AD-6 16S ribosomal RNA gene partial sequence
DQ420167.1 Gordonia shandongensis strain SD29 16S ribosomal RNA gene partial sequence
X87340.1 Gordonia hydrophobica DNA for 16S ribosomal RNA
AM406674.1 Gordonia malaquae partial 16S rRNA gene type strain IMMIB WWCC-22
AB624257.1 Gordonia iterans gene for 16S ribosomal RNA partial sequence
JN008111.1 Gordonia alkaliphila strain CJ10 16S ribosomal RNA gene partial sequence
HE574551.1 Gordonia phosphorivorans partial 16S rRNA gene type strain Ca8T
JF806526.1 Gordonia caeni strain MJ32 16S ribosomal RNA gene partial sequence
X93485.1 Gordonia hirsuta 16S rRNA gene
AF432348.1 Gordonia sp. HD321 16S ribosomal RNA gene partial sequence
X79286.1 Gordonia terrae (DSM 43249) 16S rRNA gene
LC072670.1 Gordonia hongkongensis gene for 16S rRNA partial sequence Intra-strain 16S rRNA gene copy 1 strain: HKU50
EF151959.1 Gordonia lacunae strain BS2 16S ribosomal RNA gene partial sequence
AF101416.1 Gordonia desulfuricans strain 213E 16S ribosomal RNA gene partial sequence
AF101418.1 Gordonia amicalis 16S ribosomal RNA gene partial sequence
AF380930.1 Gordonia namibiensis NAM-BN063A 16S ribosomal RNA gene partial sequence
X80632.1 Gordonia rubripertinctus 16S rRNA gene strain DSM43197T
KR819397.1 Gordonia sp. YC-RL2 16S ribosomal RNA gene partial sequence
Y18054.1 Gordonia alkanivorans 16S rRNA gene
Strain YC-RL2 (Genome derived 16S ribosomal RNA gene sequence)
AF148947.1 Gordonia nitida 16S ribosomal RNA gene partial sequence
JN615417.2 Gordonia didemni strain B204 16S ribosomal RNA gene partial sequence

Figure S1. A phylogenetic tree showing the relationship between 16S rDNA of strain YC-RL2 and 16S rDNA of validly published *Gordonia* species. The evolutionary history was inferred using the Neighbor-Joining algorithm. The bootstrap consensus tree inferred from 1000 replicates is taken to represent the evolutionary history of the taxa analyzed.

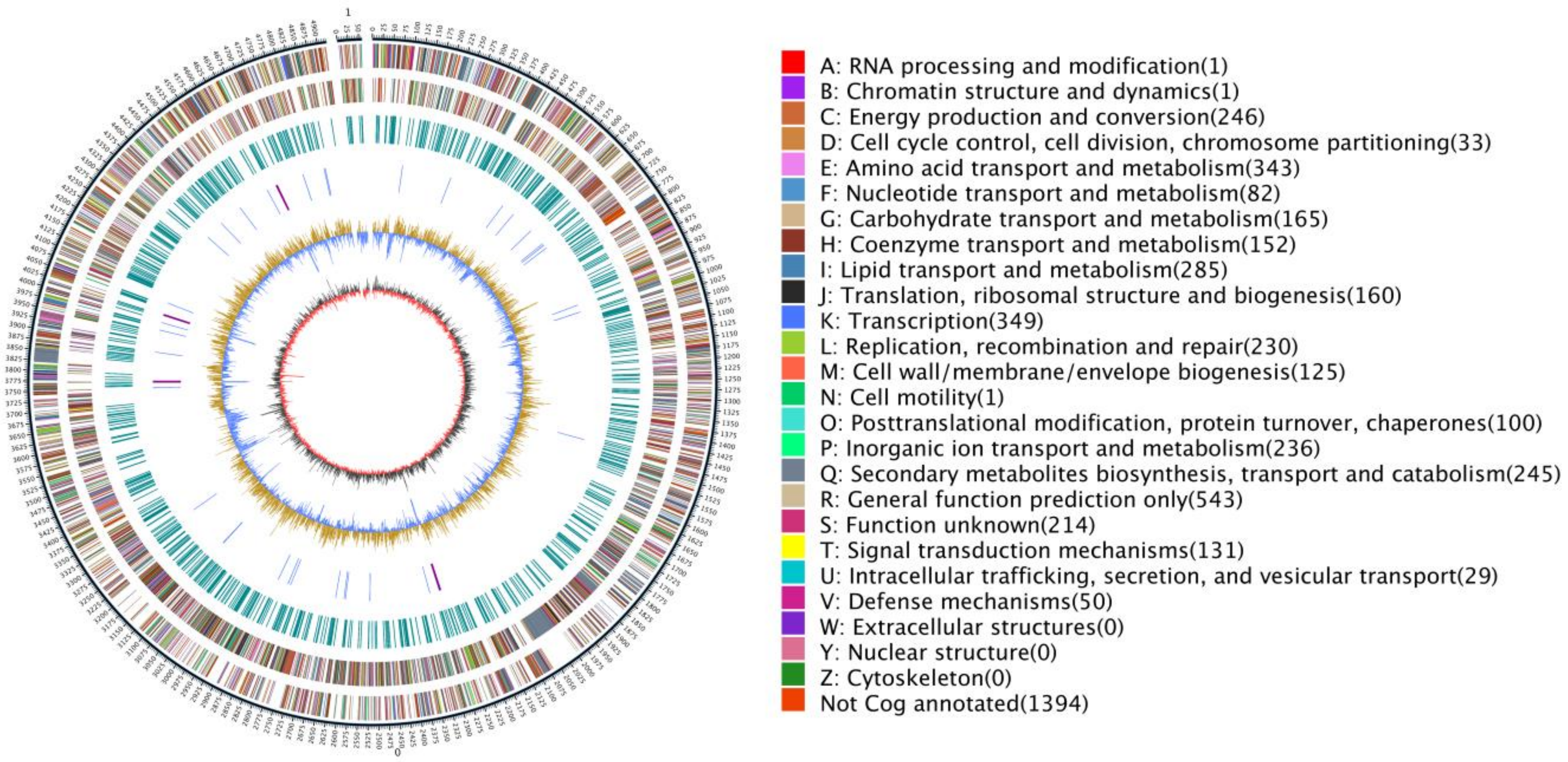

Figure S2. Genome visualization by Circos.

The outside circle is the size of the genome, each scale is 5 Kb; The second and third circles are the genes on the positive and negative strands of the genome, and different colors represent different COG functions. The fourth circle is the repeating sequence; The fifth circle is tRNA and rRNA, blue is tRNA, purple is rRNA; The sixth circle represents GC content, light yellow means that the GC content of this area

is higher than the average GC content of the genome, the higher the peak value, the greater the difference with the average GC content, blue means that the GC content of this area is lower than the average GC content of the genome, the higher the peak value, the greater the difference with the average GC content; The innermost ring is GC-skew, and the dark gray represents that G content of this region is greater than C, and red represents that C content of this region is greater than G.

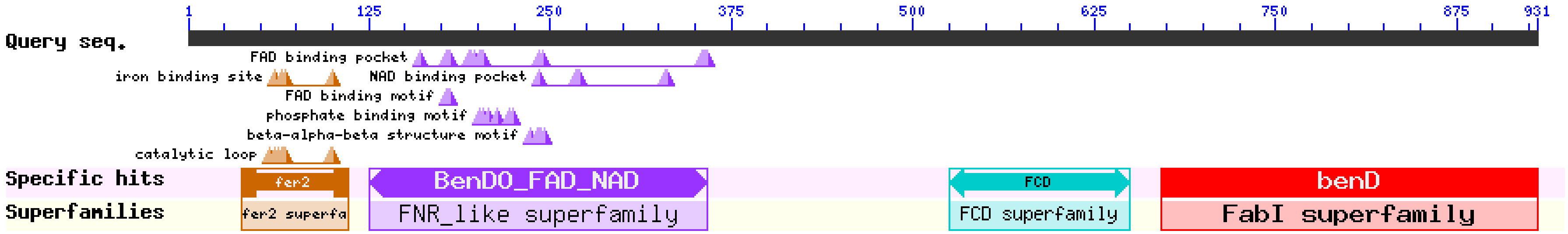

Figure S3. The conserved domains of putative *ben*CD as predicted by NCBI conserved domain database (https://www.ncbi.nlm.nih.gov/cdd)

Table S1: The Table showing conserved domains putative dioxygenases in YC-RL2 genome as predicted by SMART and CDD databases

Annotated function(NCBI) and Refseq accession	Gene location in genome (bp)	Strand	SMART		CDD	
			Domain	E-value	Domain/super family	E-value
2,3-dihydroxybiphenyl -1,2-dioxygenase<br><br>WP_006357080.1	192433..193344	-	Glyoxalase	3.2e-21	HpaD	4.5e-90
Aromatic ring-hydroxylating dioxygenase subunit alpha<br><br>WP_006358448.1	194845..196077	+	Rieske	1.8e-16	HcaE	1.6e-42
					Rieske	1.7e-71
Aromatic-ring-hydroxylating dioxygenase subunit alpha<br><br>WP_020171819.1	415169..416350	-	Rieske	6.2e-15	RHO_alpha_C_3	1.2e-66
			Ring_hydroxyl_A	6.1e-20	Rieske_RO_Alpha_N	4.8e-40
Benzoate 1,2-dioxygenase small subunit(*benB*)<br>WP_006358138.1	1608927..1609460	-	Ring_hydroxyl_B	3.3e-45	NTF2_like	1.7e-92
Benzoate 1,2-dioxygenase large subunit(*benA*)<br><br>WP_006867536.1	1609472..1610860	-	Rieske<br><br>Ring_hydroxyl_A	8.6e-20<br><br>3.9e-15	HcaE	0

Catechol 1,2-dioxygenase (*catA*)<br><br>WP_010842742.1	1613188..1614054	+	Dioxygenase_N<br><br>Dioxygenase_C	3.2e-16<br><br>3.2e-16	catachol_actin	3.74e-169
4,5-DOPA dioxygenase extradiol<br><br>WP_005198510.1	2322998..2323858	-	LigB	7.8e-35	45_DOPA_Dioxygenase	9.1e-115
Biphenyl-2,3-diol 1,2-dioxygenase (*bphC*)<br><br>WP_005198618.1	2379497..2380411	-	Glyoxalase	2.3e-24	HpaD	8.6e-140
Cysteine dioxygenases<br><br>WP_006359465.1	2423655..2424248	-	CDO_I	7e-15	cupin_like	9.8e-12
Glyoxalase/bleomycin /dioxygenases family protein<br><br>WP_006368839.1	3484312..3484806	+	Glyoxalase	1.5e-7	VOC	5.6e-54
Glyoxalase/bleomycin /dioxygenases family protein<br><br>WP_006368839.1	3808576..3809070	+	Glyoxalase	1.5e-7	VOC	5.44e-54

Glyoxalase/bleomycin /dioxygenases family protein<br><br>WP_006368839.1	3949408..3949902	-	Glyoxalase	1.5e-7	VOC	5.44e-54
catechol 1,2-dioxygenase(*catA*)<br><br>WP_020109559.1	4429156..4430034	-	Dioxygenase_N<br><br>Dioxygenase_C<br><br>CarboxypepD_re g	6.5e-15<br><br>8.3e-66<br><br>3.6e-10	PcaH	8.54e-157
Glyoxalase/bleomycin /dioxygenases family protein<br><br>WP_006867416.1	4430474..4430824	+	Glyoxalase	6.9e-10	VOC	1.11e-10
alpha-ketoglutarate-dependent dioxygenase AlkB<br><br>WP_005196692.1	4758698..4759327	-	2OG-FeII_Oxy_2	1.4e-13	AlkB	4.20e-30
taurine dioxygenases WP_006359682.1	4562046..4562957	-	TauD	6.9e-58	CAS_like	1.90e-93
Dioxygenase<br><br>WP_005195076.1	3436552..3437994	+	RPE65	1.2e-102	RPE65	1.10e-126

quercetin 2,3-dioxygenase<br><br>WP_006357080.1	1347185..1347946	-	Pirin	9.4e-29	YhaK	1.48e-42
aromatic-ring-hydroxylating dioxygenases<br><br>WP_005193785.1	1182316..1182915	-	Ring_hydroxyl_B	1.7e-28	ring_hydroxylating_dioxygenases_beta	
glyoxalase/bleomycin resistance/dioxygenase family protein<br><br>WP_006368839.1	929492..929983	-	Glyoxalase	1.3e-7	VOC	5.6e-54
homogentisate 1,2-dioxygenase<br><br>WP_006373199.1	1111136..1112245	+	-	-	HmgA	3.31e-35

Table S2: The table showing BGCs prediction based on the genome sequence of YC-RL2.

Cluster	Type	From	To	Most similar known cluster	MIBiG BGC-ID
Cluster 1	Cf_putative	56057	78531	-	-
Cluster 2	Cf_saccharide-Nrps	90464	152439	Chartreusin_biosynthetic_gene_cluster (15% of genes show similarity)	BGC0000206_c2
Cluster 3	Cf_putative	227341	237233	-	-
Cluster 4	Cf_putative	298190	310862	-	-
Cluster 5	Siderophore-Cf_saccharide-Nrps	417544	503809	Cahuitamycins_biosynthetic_gene_cluster (12% of genes show similarity)	BGC0001351_c1
Cluster 6	Cf_putative	693176	709543	-	-
Cluster 7	Cf_putative	710513	733183	-	-
Cluster 8	Cf_putative	744412	764664	-	-
Cluster 9	T1pks	766438	811780	-	-
Cluster 10	Cf_putative	850072	869373	-	-
Cluster 11	Cf_putative	1190817	1205993	-	-
Cluster 12	Bacteriocin	1210361	1264576	Nystatin_biosynthetic_gene_cluster (9% of genes show similarity)	BGC0000115_c1
Cluster 13	Terpene	1284786	1315488	Sioxanthin_biosynthetic_gene_cluster (62% of genes show similarity)	BGC0001087_c3
Cluster 14	Cf_putative	1323496	1336805	-	-
Cluster 15	Other	1386635	1430522	-	-
Cluster 16	Cf_putative	1492040	1502157	-	-
Cluster 17	Cf_putative	1538898	1550887	-	-
Cluster 18	Cf_putative	1558881	1569115	-	-
Cluster 19	Cf_putative	1573654	1586608	-	-
Cluster 20	Cf_putative	1598974	1609460	Bacillomycin_biosynthetic_gene_cluster (20% of genes show similarity)	BGC0001090_c1
Cluster 21	Cf_putative	1709028	1728950	Coelichelin_biosynthetic_gene_cluster (27% of genes show similarity)	BGC0000325_c1
Cluster 22	Cf_putative	1755055	1775100	Nataxazole_biosynthetic_gene_cluster (7% of genes show similarity)	BGC0001213_c1
Cluster 23	Cf_putative	1790442	1811897	Rifamycin_biosynthetic_gene_cluster (3% of genes show similarity)	BGC0000136_c1

Cluster 24	Nrps	1828369	1891984	-	-
Cluster 25	Cf_putative	1899210	1929508	-	-
Cluster 26	Cf_putative	1946902	1966828	-	-
Cluster 27	Nrps	1981922	2110193	Kanamycin_biosynthetic_gene_cluster (2% of genes show similarity)	BGC0000703_c1
Cluster 28	Cf_putative	2366173	2393891	-	-
Cluster 29	Cf_saccharide	2414032	2447390	-	-
Cluster 30	Cf_putative	2625112	2648675	-	-
Cluster 31	Cf_putative	2650173	2656293	-	-
Cluster 32	Cf_putative	2713365	2736276	-	-
Cluster 33	Ectoine	2888950	2899390	Ectoine_biosynthetic_gene_cluster (75% of genes show similarity)	BGC0000853_c1
Cluster 34	Cf_putative	3101016	3118082	-	-
Cluster 35	Terpene	3227709	3248845	SF2575_biosynthetic_gene_cluster (6% of genes show similarity)	BGC0000269_c1
Cluster 36	Cf_putative	3389080	3408641	-	-
Cluster 37	Arylpolyene	3437047	3478210	-	-
Cluster 38	Cf_putative	3556031	3589180	-	-
Cluster 39	Cf_putative	3701917	3717063	Paromomycin_biosynthetic_gene_cluster (5% of genes show similarity)	BGC0000712_c1
Cluster 40	Nrps	3800581	3874635	Echosides_biosynthetic_gene_cluster (11% of genes show similarity)	BGC0000340_c1
Cluster 41	Cf_fatty_acid	3949996	3985651	-	-
Cluster 42	Cf_putative	4111856	4122561	-	-
Cluster 43	Cf_putative	4225841	4238236	-	-
Cluster 44	Cf_putative	4263168	4275116	-	-
Cluster 45	Cf_putative	4277071	4282988	-	-
Cluster 46	Cf_putative	4311246	4322764	-	-
Cluster 47	Cf_putative	4347274	4373809	-	-
Cluster 48	Cf_fatty_acid	4422826	4443842	-	-
Cluster 49	Cf_putative	4444140	4464431	-	-
Cluster 50	Cf_putative	4583040	4596896	-	-
Cluster 51	Cf_fatty_acid	4604465	4625490	-	-

Cluster 52	Cf_putative	4747252	4761136	-	-
Cluster 53	Nrps	4857142	4907468	-	-
